# Supplementary material for: Tead1a Initiates Transcriptional Priming Through the TEAD1a/YAP‐Notch1‐Spi1/Cebpα Axis to Promote Neutrophil Fate
Source: Adv Sci (Weinh). 2025 Aug 28;12(41):e05441. doi: 10.1002/advs.202505441 (PMC12591127; doi:10.1002/advs.202505441)
Supplement: Supplementary file 4 — Supplementary Dataset [file ADVS-12-e05441-s004.zip › 3-7.zip!/3-7/02-7.cluster/cluster18/Enrich/ego_BP.pdf]

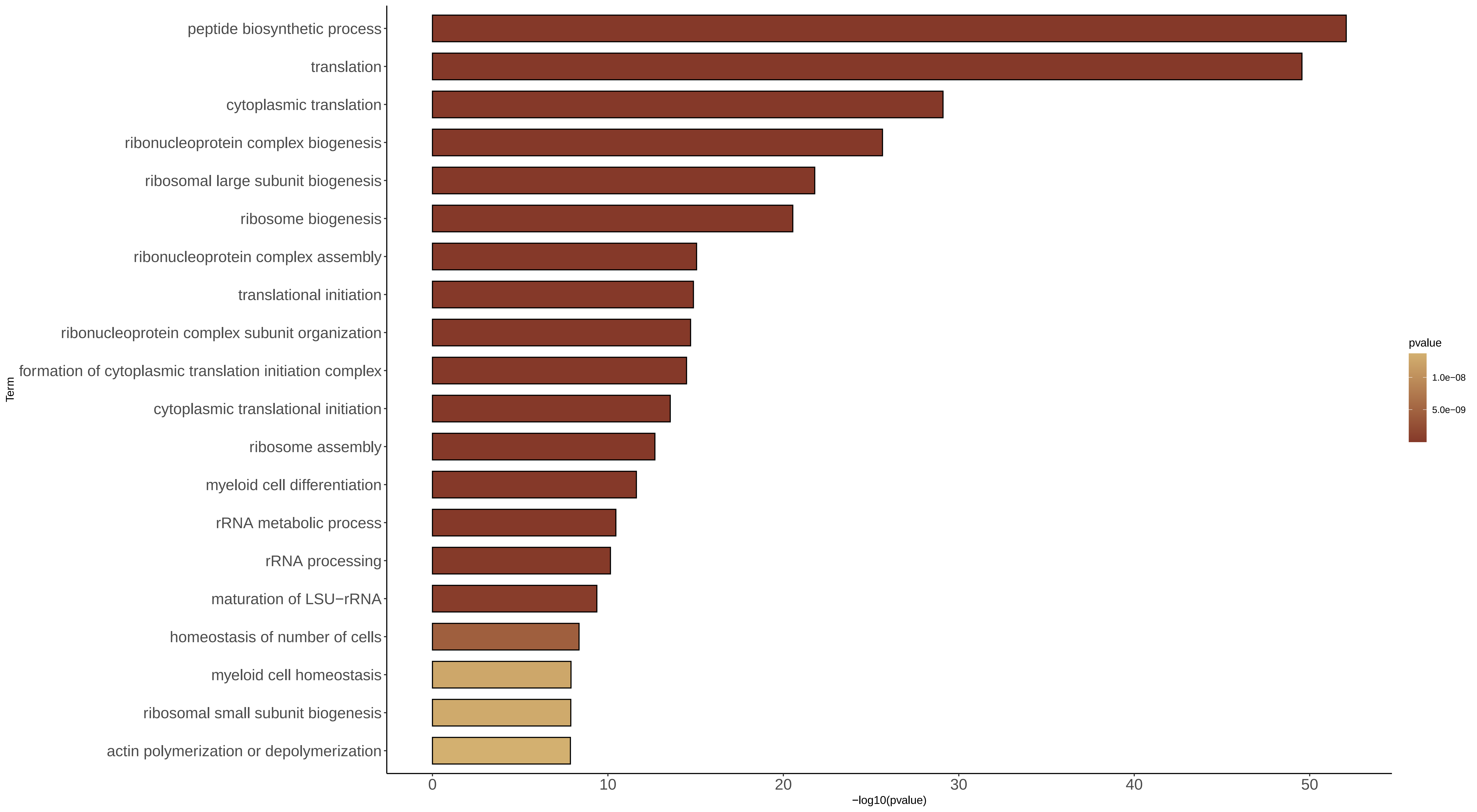

peptide biosynthetic process
translation
cytoplasmic translation
ribonucleoprotein complex biogenesis
ribosomal large subunit biogenesis
ribosome biogenesis
ribonucleoprotein complex assembly
translational initiation
ribonucleoprotein complex subunit organization
formation of cytoplasmic translation initiation complex
cytoplasmic translational initiation
ribosome assembly
myeloid cell differentiation
rRNA metabolic process
rRNA processing
maturation of LSU−rRNA
homeostasis of number of cells
myeloid cell homeostasis
ribosomal small subunit biogenesis
actin polymerization or depolymerization
Term
0
10
20
30
40
50
−log10(pvalue)
pvalue
1.0e−08
5.0e−09
